# Antiangiogenic and Antioxidant In Vitro Properties of Hydroethanolic Extract from açaí (*Euterpe oleracea*) Dietary Powder Supplement

**DOI:** 10.3390/molecules26072011

**Published:** 2021-04-01

**Authors:** Raquel Costa, Daniela Azevedo, Pedro Barata, Raquel Soares, Luís F. Guido, Daniel O. Carvalho

**Affiliations:** 1 i3S-Instituto de Investigação e Inovação em Saúde, Universidade do Porto, 4200-135 Porto, Portugal; raquel.costa@i3s.up.pt (R.C.); pbarata@ufp.edu.pt (P.B.); raqsoa@med.up.pt (R.S.); 2 Departamento de Biomedicina, Unidade de Bioquímica, Faculdade de Medicina, Universidade do Porto, 4200-319 Porto, Portugal; up201504492@fc.up.pt; 3 REQUIMTE/LAQV, Departamento de Química e Bioquímica, Faculdade de Ciências, Universidade do Porto, Rua do Campo Alegre 687, 4169-007 Porto, Portugal; lfguido@fc.up.pt; 4 Faculdade de Ciências da Saúde, Universidade Fernando Pessoa, Praça 9 de Abril 349, 4249-004 Porto, Portugal

**Keywords:** *Euterpe oleracea*, açaí, phenolic compounds, anthocyanins, antioxidants, angiogenesis HPLC-DAD-ESI-MS^n^

## Abstract

The *Euterpe oleracea* fruit (açaí) is a promising source of polyphenols with health-promoting properties. To our knowledge, few studies have focused on the influence of açaí phytochemicals on angiogenesis, with a significant impact on cancer. This study aimed at investigating the phytochemical profile of a purple açaí hydroethanolic extract (AHE) obtained from a commercial dietary powder supplement by high-performance liquid chromatography coupled to diode array detection and electrospray ionization mass spectrometry, and evaluate its in vitro effects on distinct angiogenic steps during vessel growth and on oxidative markers in human microvascular endothelial cells (HMEC-1). The phenolic profile of AHE revealed the presence of significant levels of anthocyanins, mainly cyanidin-3-*O*-rutinoside, and other flavonoids with promising health effects. The in vitro studies demonstrated that AHE exerts antiangiogenic activity with no cytotoxic effect. The AHE was able to decrease HMEC-1 migration and invasion potential, as well as to inhibit the formation of capillary-like structures. Additionally, AHE increased antioxidant defenses by upregulating superoxide dismutase and catalase enzymatic activities, accompanied by a reduction in the production of reactive oxygen species. These data bring new insights into the potential application of angiogenic inhibitors present in AHE on the development of novel therapeutic approaches for angiogenesis-dependent diseases.

## 1. Introduction

The açaí (*Euterpe olerácea* Mart.) is a palm tree native to South America and mainly found along the Amazon River estuary, Brazil, Guyana and Venezuela. The açaí fruit, a small and round dark purple berry, gained a great commercial interest in the past years due to its nutritional and functional properties. According to in vitro and in vivo studies, it has been associated with relevant biological activities such as antioxidant and anti-inflammatory properties, as well as anti-carcinogenic, antimicrobial, anti-diabetic, anti-neurodegenerative, and cardio-protective effects [1,2].

Some studies have demonstrated that açaí fruit exhibits antiangiogenic and anti-inflammatory behavior responsible for its antitumoral effect [3,4]. However, the endothelial mechanisms responsible for the modulation of vessel growth have not yet been elucidated. Angiogenesis consists in the growth of new blood vessels from preexistent vasculature and it is one of the key players underlying these processes [5]. Angiogenesis is essential both in physiological conditions, namely during development, tissue repair and reproduction, and in a range of angiogenesis-dependent diseases as cancer, chronic metabolic diseases and cardiovascular diseases. Angiogenic process is a rate-limiting step during tumor growth and metastasis formation, as tumor cells constitute an active source of angiogenic stimuli, as vascular endothelial growth factor (VEGF-A) and hypoxia [5]. The process comprises several steps including the degradation of the vessel adjacent basement membrane, detachment of the surrounding pericytes and remodeling of the involving extracellular matrix, allowing endothelial cells migration, invasion, proliferation and assembly into tubular structures, promoting endothelial cell anastomosis and vessel branching to create new angiogenic vessels [6].

As revised by Yamaguchi et al., the açaí pulp and seed extracts revealed high levels of antioxidants and bioactive phenolic compounds, such as anthocyanins and other flavonoids (e.g., orientin, isovitexin, scoparin and taxifolin deoxyhexose) [1]. The antioxidant capacity of açaí extracts has been mainly associated with their high content and composition in anthocyanins, water-soluble pigments responsible for the intense purple color of the fruit. The cyanidin-3-*O*-rutinoside has been identified as the most predominant anthocyanin in purple açaí, followed by cyanidin-3-glycoside and traces of other cyanidin, peonidine and pelargonidin derivatives [7,8,9,10,11,12,13,14].

Nowadays, the dietary sources of antioxidants have been increasingly encouraged in human diet in the context of a healthier lifestyle. Though, the açaí pulp degrades rapidly at room temperature and it is easily prone to post-harvest contaminations [15]. For this reason, the interest on the development of açaí berry dietary supplements (e.g., capsules, lyophilized powder and liquid concentrates) has emerged, in order to reduce the degradation of bioactive compounds during transportation and storage, and even to be incorporated into foods and beverages [1]. However, few information is available about the phenolic composition and health benefits of commercially available dietary açaí supplements, and the impact of industrial processing on bioactive compounds. The comparison of different açaí dietary supplements has demonstrated that they may contain lower contents of phenolic compounds, or even no detectable anthocyanins comparing to non-commercial açaí fruit [14,16]. According to Xiong et al., industrially processed fruit exhibited lower proanthocyanidins content (up to approx. 83%) but higher levels of anthocyanins (20-fold higher) comparing to non-commercial açaí [17].

The present study aimed to investigate the phenolic profile of a purple açaí hydroethanolic extract (AHE) obtained from a commercial dietary powder supplement by high-performance liquid chromatography coupled to diode array detection and electrospray ionization tandem mass spectrometry (HPLC-DAD-ESI-MS^n^), and evaluate its potential antioxidant and antiangiogenic mechanisms in human microvascular endothelial cells (HMEC-1). The açaí is mainly commercialized as a powder in many countries, so there is a significant interest in studying this type of dietary supplement as a source of bioactive compounds with health benefits and potential applications as a functional ingredient.

## 2. Results and Discussion

### 2.1. Phenolic Profile of AHE by HPLC-DAD-ESI-MS^n^

**Figure 1:**
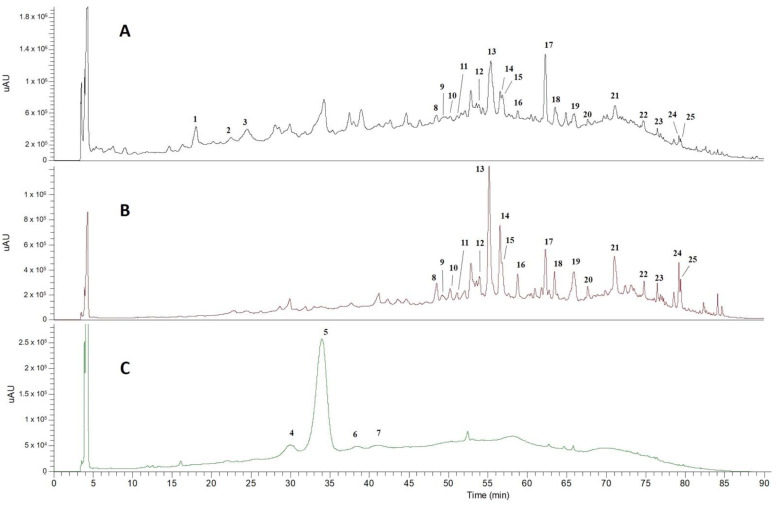
Chromatograms of açaí hydroethanolic extract (AHE) obtained at 280 nm (**A**), 340 (**B**) and 520 (**C**). Identified signals are presented in Table 1 and Table 2.

The main phytochemicals present in AHE were tentatively identified by HPLC-DAD-ESI-MS^n^. As presented in Figure 1, the obtained chromatograms allowed the identification of 25 phenolic compounds using positive and negative ionization modes, namely anthocyanins and other flavonoids. The tentatively identified compounds are summarized in Table 1 and Table 2, and were identified based on bibliographic references, MassBank data and by comparison with standards MS and UV spectra.

The chromatographic profile of AHE acquired at 520 nm (Figure 1C) revealed the presence of four main compounds (Peaks 4, 5, 6 and 7 at Rt = 30.6, 34.8, 38.7 and 41.2 min, respectively). These compounds were undoubtedly identified as anthocyanins by exhibiting a characteristic maximum absorption wavelength at 520 nm and responsible for the intense purple pigmentation of açaí. The MS analysis was conducted in positive ionization mode since it allowed a better ionization and the selective detection of these compounds (Compounds **4**–**7**, Table 1).

The compounds represented by Peaks 4 and 5 (Figure 1C) were tentatively identified as cyanidin glycoside derivatives, characterized by major ion signals at *m*/*z* 287 (cyanidin) (Table 1). According to literature, Compounds **4** and **5** were tentatively identified as cyanidin-3-*O*-rutinoside and cyanidin-3-*O*-glucoside, respectively [8,9,10,11,18,19,20]. The most abundant anthocyanin identified in AHE was cyanidin-3-*O*-rutinoside, corresponding to 89 ± 0.3% of the total anthocyanins extracted from the açaí dietary supplement (Table 1). In agreement, the identified cyanidin derivatives were also described in high contents in commercial and non-commercial freeze-dried açaí berries, with predominance of cyanidin-3-*O*-rutinoside [7,8,9,10,11,13,14,19].

Two other anthocyanins were identified in AHE (Figure 1C, Peaks 6 and 7). Peak 6 (Rt = 38.7 min) exhibited a parent ion at *m*/*z* 609 and an intense ion at *m*/*z* 301 in the MS^2^ spectra, corresponding to the anthocyanidin peonidin (Table 1). The compound was tentatively identified as peonidin-3-*O*-rutinoside (2.6 ± 0.5% of total anthocyanin content) as previously described in açaí fruits from Brazil [8,9,20] and Colombia [11]. Based on previous information published by Garzon et al., Peak 7 (Rt = 41.2 min) was tentatively identified as pelargonidin-3-*O*-rutinoside (2.3 ± 0.1% of total anthocyanin content), and identified in low content in fully ripe açaí pulp (bellow 1% of total anthocyanins content) [11]. In comparison, cyanidin-3-*O*-rutinoside was identified as the predominant anthocyanin in different açaí samples (up to 100% of the total anthocyanins content), followed by cyanidin-3-*O*-glucoside (up to 19%) and peonidin 3-*O*-rutinoside (up to 4%) [7,8,9,10,11]. According to this information, the anthocyanins profile of AHE obtained from açaí powder supplement (Figure 1C, Table 1) was found to be very similar to that described for fresh açaí.

A diversity of other non-anthocyanin phenolic compounds was identified in AHE (Table 2).

Peak 1 (Figure 1, Rt = 18.2 min) was tentatively identified as protocatechuic acid (dihydroxybenzoic acid) by comparison with commercial standard. The content of protocatechuic acid in AHE (9.4 ± 0.9 mg/100 g dw) was significantly higher comparing to the contents described in the fresh fruit (up to 1.7 mg/100 g dw) [11,13,19].

Peaks 2 and 3 (Figure 1, Rt = 22.5 min and 23.5 min) exhibited pseudomolecular ions at *m*/*z* 865 and 577, respectively (Table 2). These compounds were identified as oligomers of flavan-3-ols (proanthocyanidins). A detailed inspection of the mass spectra of Compound **1** allowed to identify characteristic fragments of a proanthocyanidin trimer. In association, Peak 2 was identified as a dimeric proanthocyanidin [21,22]. According to previous works, proanthocyanidins have been described as one of the major phytochemicals in commercial and non-commercial açaí samples [9,10,17,19]. Recently, Martins et al. discovered that B-type and A-type procyanidins were also among the major components in açaí seeds [22]. A detailed inspection of the MS profile of the proanthocyanidins in AHE allowed to conclude that the identified oligomeric forms in the powder supplement were predominantly B-type procyanidins [22]. In our work, the content of proanthocyanidins in AHE were found to be between 6.3 and 25.7 mg catechin/100 g dw. In comparison, the content of procyanidins in de-seeded fully ripe fruits were reported between 0.7 and 5.3 mg catechin/kg [19].

A further investigation of the phenolic compounds in AHE revealed the presence of different flavonoids classes and their corresponding *C*- and *O*-glycosylated forms (Figure 1A,B; Table 2). The characterization of AHE revealed the presence of flavones as one of the main classes of flavonoids. Peaks 8, 12, 16, 20 and 21 (Figure 1A,B) were tentatively identified as *C*-glycoside conjugates of apigenin (Table 2) by the characteristic fragments of *C*-glycosylated and di-*C*-glycosylated flavonoids [23]. The concentration of apigenin derivatives in AHE ranged between 4.1 and 11.6 mg rutin/100 g dw. In particular, apigenin-6,8-di-*C*-pentoside has not been previously reported in fresh açaí fruit [11,18,19,24], while apigenin-di-*C*-hexoside sulfate was reported in leaflet extracts of *Euterpe oleracea* [25].

The flavone luteolin was also identified in AHE (Peak 23, Rt = 77.2 min) by a molecular ion at *m*/*z* 285 (Table 2) and a characteristic fragmentation pattern in MS^2^ spectra [18]. Peaks 9, 10, 11 (Rt = 49.6, 50.6 and 51.4 min., respectively) showed a pseudomolecular ion at *m*/*z* 579 and the MS^n^ spectra revealed characteristic fragment ions of a di-*C*-asymmetric glycosylated flavone. The ions at *m*/*z* 399 [aglycone + 113]^−^ and 369 [aglycone + 83]^−^ support the aglycone luteoline [23]. Thus, the compounds were tentatively identified luteolin-*C*-hexoside-*C*-pentoside isomers (Table 2). Peak 19 revealed a parental ion at *m*/*z* 689 and a very similar fragmentation pattern comparing to the compounds described previously (Peaks 9–11). However, the obtained information was not enough to propose a reliable identification. Peaks 14 and 15 (Tr = 56.9 and 57.3 min, respectively) were attributed to luteolin-*C*-hexose isomers orientin and isoorientin, respectively, which have been already reported in açaí fruit [7,11,19,24], juice [18,26] and freeze-dried powders [13]. The content of orientin and isoorientin in AHE (12.7 ± 0.5 and 5.4 ± 0.1 mg rutin/100 g dw, respectively) obtained from açaí dietary powder were found to be similar comparing to the previously described contents in açaí pulp (15 and 10 mg rutin equivalents/100 g dw for orientin and isoorientin, respectively) [11], but around 6-fold lower comparing to the levels reported in other açaí powder dietary supplements [16]. According to Gordon et al., the content of orientin and isoorientin is largely affected by fruit maturation stage and significantly decreases during ripening [13].

Peak 18 (Tr = 64.1 min) was also identified as a glycosylated flavone with a deprotonated molecular ion at *m*/*z* 461 in the mass spectrum. The fragments at *m*/*z* 371 [M − H − 90]^−^ and 341 [M − H − 120]^−^ are characteristic of scoparin [10,11,18,19].

Peak 25 was tentatively identified as the *O-*methylated flavone tricin, with [M − H]^−^ at *m*/*z* 329 and characteristic fragments at *m*/*z* 314 and 299 [27]. According to previous studies, the presence of tricin has not been reported in fresh açaí [11,18,19].

Two main flavanonols were identified in AHE by HPLC-DAD-ESI-MS^n^. One of the most intense peaks identified in the chromatogram at 340 nm (Figure 1B, Peak 13, Rt = 55.8 min), showed a deprotonated molecule at *m*/*z* 449 and the MS^n^ spectra revealed characteristic fragment ions of the flavanonol taxifolin (Table 2). The compound was identified as taxifolin deoxyhexose, one of the most abundant non-anthocyanin phenolics identified in açaí berries [11,19]. The concentration of taxifolin deoxyhexose in AHE (33.1 ± 0.5 mg rutin/100 g dw) was found to be significantly higher comparing to previously described contents in açaí fresh berries (up to 2.8 mg rutin/100 g dw) [11]. The compound corresponding to Peak 17 (Tr = 62.7) was attributed to dihydrokaempferol, identified in açaí fruits from Colombia [11] and Brazil [18,24]. Its concentration in AHE was found to be around 9.6 ± 0.1 mg rutin/100 g dw, comparing to concentrations ranging between 0.3 and 0.5 mg rutin/100 g dw in açaí pulp [11].

Flavonols were also identified in AHE (Table 2, Peaks 22 and 24). The Compound **22** (Figure 1A,B, Rt = 75.4 min) showed a fragmentation pattern consistent with quercetin (Table 2) and was previously identified in açaí juice [18] and in freeze-dried açaí powders [28]. The compound represented by Peak 24 (Figure 1) exhibited a pseudomolecular ion at *m*/*z* 315 and a characteristic fragment ion at *m*/*z* 300 in the MS^2^ spectra (Table 2). The MS^3^ spectra with a base peak at *m*/*z* 271 and other typical ions at 283, 227 and 151 allowed to infer about the presence of isorhamnetin [27]. In contrast, Garzon et al. only reported the presence of isorhamnetin-rutinoside in fully ripe açaí pulp [11].

As revealed, it was found that AHE obtained from an açaí dietary powder supplement is an excellent source of bioactive phenolic compounds associated with a broad spectrum of health beneficial effects [1]. Despite the increasing number of studies focused on açaí bioactivity, little is known regarding its angiogenic potential. We have been addressing the in vitro and in vivo effects of diet polyphenols on angiogenesis. We showed that different classes of phenolic compounds (e.g., beer-derived, coffee and raspberry phenolic compounds) decreased angiogenesis by targeting specific molecular pathways namely VEGF-A, and the underlying signaling pathway, both in physiological and pathological conditions [29,30,31]. Therefore, the potential in vitro antiangiogenic and antioxidant properties of the phenolic rich AHE were then evaluated.

### 2.2. Effect of AHE on Endothelial Cell Viability and Its Antiproliferative Behavior

In the present study, HMEC-1 treated with AHE were used to evaluate in vitro angiogenic individual steps during vessel sprouting.

**Figure 2:**
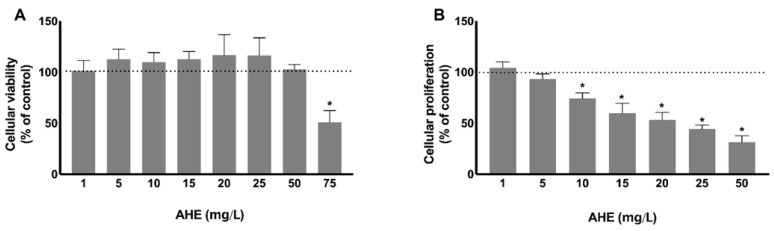
Cytotoxicity and antiproliferative capacity were assessed in HMEC-1 treated with different concentrations of AHE during 24 h. Metabolic activity was determined by MTS assay (**A**). AHE induces cytotoxicity at the highest concentration tested (75 mg/L), in comparison to control. Proliferation was evaluated by BrdU incorporation (**B**). There is an inhibition on HMEC-1 proliferation after treatment with AHE, in a dose-dependent manner. Results are expressed as % of control group. * *p* < 0.05 vs. control.

The bioactivity of AHE was evaluated on HMEC-1 by studying its possible cytotoxicity effect using MTS (3-[4,5-dimethylthiazol-2-yl]-5-[3-carboxymethoxy-phenyl]-2-[4-sulfophenyl]-2H-tetrazolium) viability assay. As depicted in Figure 2A, our results showed that AHE did not exert cytotoxic effects at lower doses, but reduced almost 50% of mitochondrial activity at the highest concentration tested (75 mg/L), affecting cellular viability, in comparison to control group (*p* = 0.0026). In accordance, it has been recently demonstrated that açaí oil induces hepatic and thyroid tissue toxicity in male Wistar rats, after the intake of oil doses higher than 100 mg/kg for 14 days [32]. In comparison, no genotoxic effects were reported for the same doses in leukocytes, liver, bone marrow and testicular rat cells [33]. The toxic effects registered by histological and histochemical analysis were attributed to palmitic and oleic acids, major constituents of açaí oil, together with vanillic, g-linolenic, linoleic, cinnamic, caffeic, protocatechuic, ferulic, syringic acids, and flavonoids quercetin and kaempferol rutinoside [32,33]. For this reason, the further evaluation of the bioactive properties of AHE was assessed using non-toxic concentrations, in order to consider its pharmacological potential, since some of the compounds reported in fruit oil were also identified in AHE (e.g., protocatechuic acid and quercetin).

Then, the influence of the extract on the proliferative capacity was assessed by the BrdU incorporation assay in HMEC-1 incubated with different nontoxic concentrations (1–50 mg/L). Cell proliferation was diminished in a dose-dependent manner. At doses of 10 mg/L (with a mean decrease of 25.9%; *p* = 0.0005) or higher, a significant decrease in proliferation was observed, when compared with control (Figure 2B). These findings reveal that AHE inhibits endothelial cell proliferative capacity, one major step of the angiogenic process.

### 2.3. AHE Decreases Migration and Invasion Potential of Endothelial Cells

**Figure 3:**
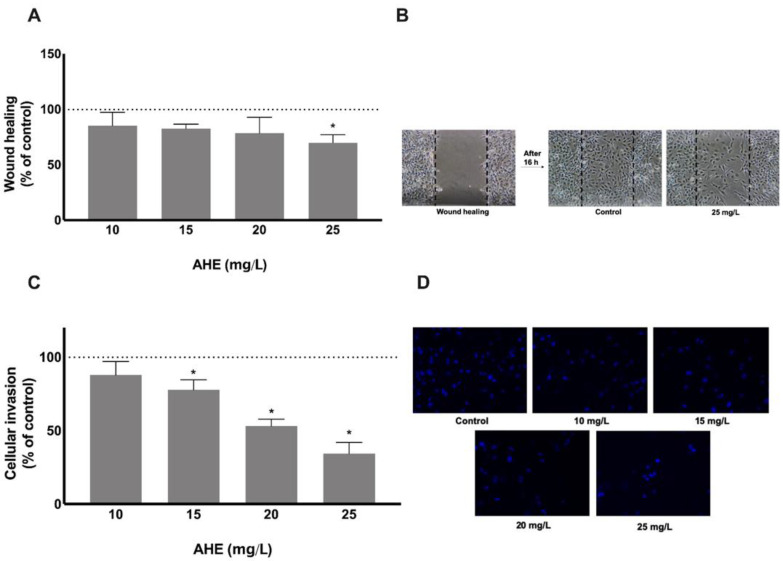
The ability to impair HMEC-1 migration and invasion was performed after 16 h and 24 h treatment with AHE by injury assay (**A**,**B**) and matrigel-coated transwell assay (**C**,**D**), respectively. There is an abrogation in cell motility and the capacity to migrate to the wound area was reduced at a concentration of 25 mg/L (**A**). Dotted lines represent the wound healing area at the beginning of experiment (**B**). HMEC-1 invasive capacity through a matrigel membrane was reduced in a dose-dependent manner with significance for doses of 15 mg/L or higher (**C**). Representative images of invasive HMEC-1 under fluorescence microscope (**D**). Results are presented as % of control group mean ± SD. * *p* < 0.05 vs. control.

During angiogenesis, cell motility and migration together with extracellular matrix invasion capacity are crucial steps to ensure vessel growth. The influence of AHE on endothelium migration and invasion capacities by wound healing assay and double chamber assay, respectively, was determined after treatment with non-cytotoxic extract concentrations ranging from 10 to 25 mg/L (Figure 3A,B). Overnight exposure revealed a decrease in HMEC-1 migration to the injured area, being significant at 25 mg/L AHE with a reduction of 30.3% (*p* = 0.0079). This migration inhibitory effect of AHE was accompanied by a reduced capacity of HMEC-1 to invade the extracellular membrane as revealed by the invasion assay.

Accordingly, as shown in Figure 3C,D, when endothelial cells were harvested on a Matrigel-coated transwell permeable chamber and the different treatments were placed on wells below the cell permeable membrane, a dose-dependent effect on the invasion capacity was denoted. A mean reduction of 22.27% (*p* = 0.0178), 46.90% (*p* < 0.0001) and 65.67% (*p* < 0.0001) at 15, 20, and 25 mg/L extract concentrations, respectively, was found when compared with control group. These findings indicate that non-cytotoxic concentrations of AHE target migration and invasiveness capacity of HMEC-1, two major features of the angiogenic process.

### 2.4. AHE Inhibited the Formation of Capillary-Like Structures Formation

**Figure 4:**
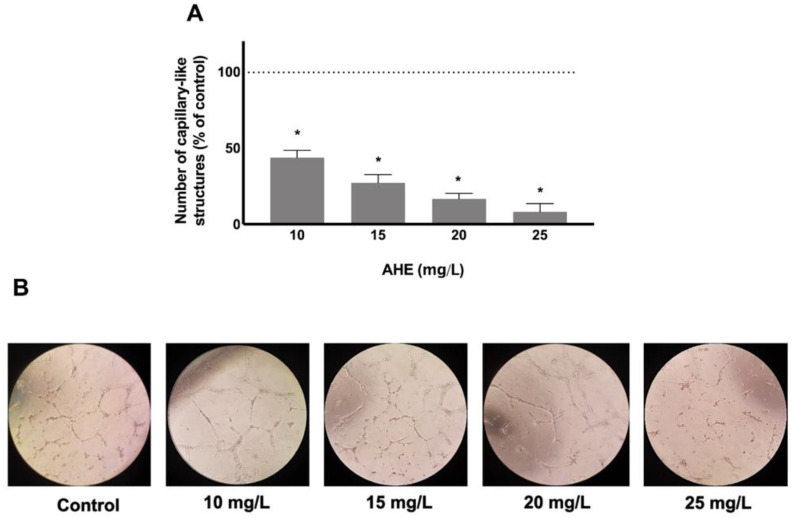
Formation of capillary-like structures was quantified in HMEC-1 after treatment with AHE or control, by Matrigel assay. HMEC-1 reduces, dose-dependently, its ability to assemble into tubular structures in concentrations ranging from 10 to 25 mg/L (**A**). Representative images of endothelial assembly after 16 h of standard treatments. (**B**) Results are expressed as % of control group. * *p* < 0.05 vs. control.

The effects of AHE on the ability of endothelial cells to assemble into capillary-like structures was also evaluated. As represented in Figure 4A,B, endothelial cells capacity to assemble into capillary-like structures and the number of branching points were significantly decreased in a dose-dependent manner for all tested conditions with unconnected structures and loose edges, when compared to control cells that exhibited well-organized and connected tubular structures. The formation of capillary-like structures was reduced between 56.28% and 91.82% for AHE concentrations of 10 and 25 mg/L, respectively (*p* < 0.0001 vs. control) (Figure 4A,B).

Taken together, our findings demonstrate the antiangiogenic properties of AHE through its effect on fundamental steps of vessel growth. Previous studies on açaí have shown its ability to suppress cancer due to antiangiogenic and anti-inflammatory actions. Alessandra-Perini and colleagues demonstrated a reduction of angiogenic markers, as VEGF and its receptor (VEGFR-2), together with a decrease in pro-inflammatory molecules and activated macrophages in an animal model of breast cancer, treated with 200 mg/Kg of açaí extract, for 16 weeks [4]. Machado and collaborators also demonstrated an antiangiogenic potential of an AHE at the same dosage, using an animal model of experimental endometriosis. There was a reduction on VEGF levels and metalloproteinase-9 expression, involved in the degradation of basal membrane [3].

### 2.5. Antioxidant Potential of AHE on Human Microvascular Endothelial Cells

Angiogenesis and oxidative stress are intermingled processes and constitute important targets for therapeutic agents, as is the case of natural polyphenols, against cancer, metabolic and cardiovascular diseases, among other pathological conditions.

**Figure 5:**
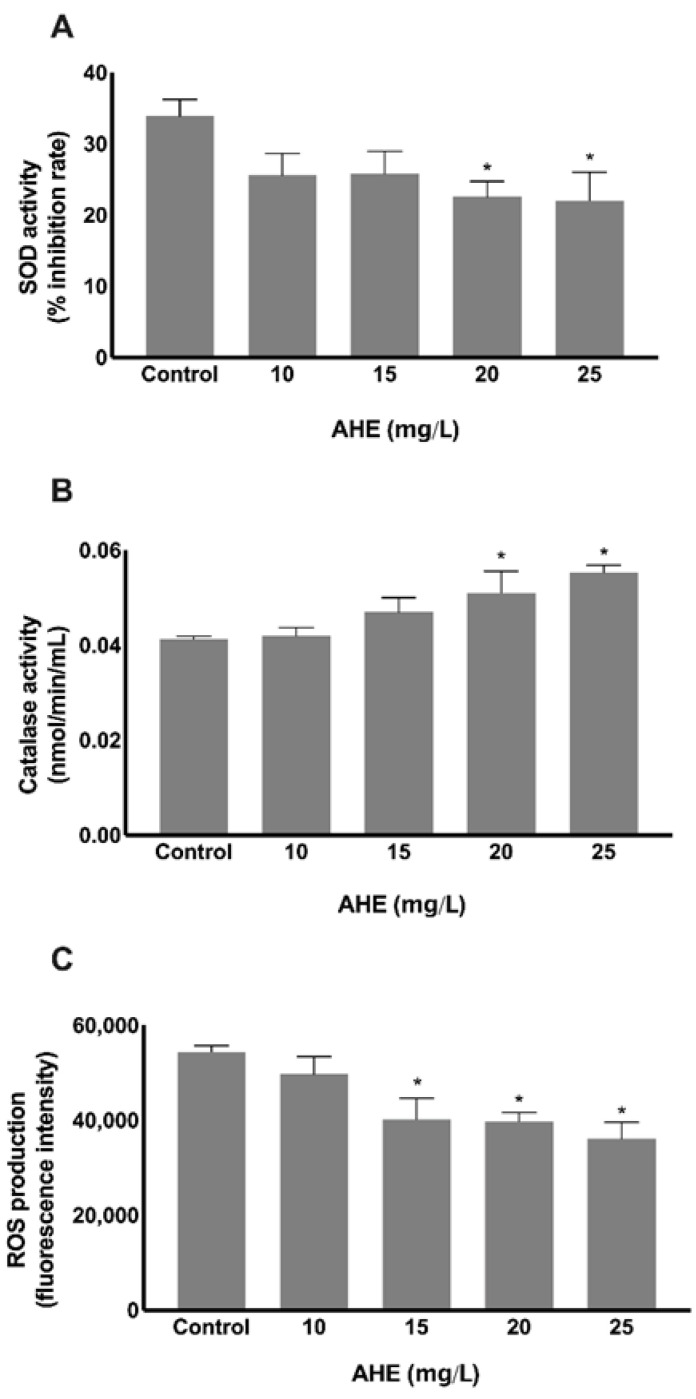
Evaluation of oxidative stress mediators after HMEC-1 treatment with AHE or control. Antioxidant SOD (**A**) and catalase (**B**) defenses were both augmented at concentrations of 20 and 25 mg/L, in comparison with control group, analyzed by enzymatic assays. Production of reactive oxygen species (ROS) was reduced dose-dependently, measured after incubation with fluorescence dies, with statistical significance at 15 mg/L or higher (**C**). Results are presented as % inhibition rate of SOD activity (**A**) nmol of catalase activity/min/mL (**B**) and units of fluorescence intensity for ROS production (**C**) * *p* < 0.05 vs. control.

In order to assess oxidative stress, superoxide dismutase (SOD) and catalase enzymes were quantified due to their importance as endogenous antioxidant defense mechanisms. As illustrated in Figure 5A, SOD activity increased when HMEC-1 were treated with AHE. A significant reduction in the percentage of enzymatic inhibition rate was identified in cells treated with 20 mg/L (23 ± 2%; *p* = 0.0094) and 25 mg/L (22 ± 4%; *p* = 0.0068) of AHE, in comparison to control group (33.90 ± 2.36%). Additionally, a dose-dependent increase in the catalase enzymatic activity was observed, as shown in Figure 5B. AHE concentrations of 20 mg/L (0.051 ± 0.005 nmol/min/mL; *p* = 0.0127) and 25 mg/L (0.055 ± 0.002 nmol/min/mL; *p* = 0.0008) increased catalase antioxidant capacity in HMEC-1 versus control group (0.041 ± 0.001 nmol/min/mL).

Oxidative stress arises as a result of excessive accumulation of reactive oxygen species (ROS) and reactive nitrogen species (RNS) in the organism, leading to irreversible cellular damage compromising the normal tissue function. In order to evaluate AHE antioxidant properties, a quantification of ROS production was addressed. A dose-dependent reduction on ROS secretion by HMEC-1 was observed upon treatment with AHE doses higher than 15 mg/L (Figure 5C). Cells treated with 15 mg/L AHE produced significant lower ROS units (40,204 ± 4449, *p* = 0.0010) in comparison to control (54,446 ± 1283 ROS units). A more pronounced antioxidant effect was registered when cells were incubated with 20 mg/L AHE (39,755 ± 1903 ROS units; *p* = 0.0014) and 25 mg/L AHE (36,115 ± 3522 ROS units; *p* < 0.0001).

Based on the obtained results, SOD and catalase enzymatic activities were increased and ROS production decreased upon AHE treatment. AHE stimulates important antioxidant defenses and reduces free radical levels. Identical achievements were also stated by Schauss et al., reporting an inhibition of ROS formation and a strong SOD activity using human neutrophils, and demonstrating that açaí constituents exert oxygen quenching function inside human cells [34]. Recently, it was found that the administration of an aqueous açaí extract (3 g/Kg) during six weeks in mice with non-alcoholic fatty liver disease also improves SOD and catalase activities together with an activation of glutathione reductase [35]. Other studies have associated the consumption of açaí juice with a higher blood antioxidant capacity in healthy volunteers. The consumption of açaí juice by healthy volunteers induced a rapid increase in the systemic antioxidant activity assessed by the cell-based antioxidant protection in erythrocytes assay, with a significant reduction in oxidative damaged within the tissues (e.g., lipid peroxidation) [36] as well as a significant increase of antioxidant enzymes activity (e.g., SOD, catalase and glutathione peroxidase) [37]. In line with these findings, another pilot study reported an increase in blood antioxidant status due to an augment not only on the activity of SOD and catalase but also in other antioxidant enzymes activity (e.g., glutathione peroxidase and reductase) and non-enzymatic oxidant defenses (glutathione), after consumption of an açaí berry-based juice blend for six weeks [38]. Similar achievements were reported in another nutritional intervention study based on the diet supplementation with açaí pulp for 4 weeks. The authors described an increase in catalase and SOD activities and glutathione levels, accompanied by a reduction on ROS production in polymorphonuclear cells, as well as an increment of serum sulfhydryl groups concentration (a marker of antioxidant capacity) [39].

The oxidative protective effects here described may be attributed to the presence of anthocyanins in AHE, predominantly cyanidin-3-*O*-rutoside and cyanidin-3-*O*-glucoside, as found by Jensen et al. [36]. In fact, anthocyanins have been described as the main contributors for açaí antioxidant properties [7,12,26,40,41]. Açaí pulp extracts, rich in anthocyanins, also exhibit health beneficial properties (e.g., neuroprotective and cardioprotective) by mechanisms dependent on their antioxidant properties [42,43,44]. Both cyanidin-3-*O*-rutinoside and cyanidin-3-*O*-glucoside, identified as the main anthocyanins in AHE, have demonstrated protective effects on neuroblastoma SH-SY5Y cells [44] and reduced necrotic glial cell death in oxidative stress conditions [45].

## 3. Materials and Methods

### 3.1. Chemicals

Rutin hydrate (95%), (+)-catechin hydrate (98%) and protocatechuic acid (97%), used in chromatographic analysis, were purchased from Sigma (St. Louis, MO, USA). High-purity water was obtained from a Direct-Q 3 UV water purification system (Millipore Iberian, Spain) and used for all analyses and glassware washing. Ethanol (EtOH, 96%) for the extractions was purchased from Chem-Lab (Zedelgem, Belgium). Methanol (MeOH) for (ultra-high pressure liquid chromatography) UHPLC Supergradient was obtained from PanReac AppliChem (Darmstadt, Germany). Formic acid (LiChropur™, 98%) used in chromatographic and mass spectrometry analyses was purchased from Sigma-Aldrich (St. Louis, MO, USA). The culture medium RPMI 1640 and all reagents used in biological assays were also purchased from Sigma-Aldrich with the maximum purity available. RIPA (Radio-Immunoprecipitation Assay) buffer was from Chemicon International (Temecula, CA, USA). All other reagents used were of analytical grade quality or higher.

### 3.2. Sample and Extract Preparation

The commercial organic *açaí* fruit powder was obtained directly from the producer (Lisboa, Portugal) and freeze-dried after reception in order to remove any residual water content. Extraction was performed by mixing 1.0 g of freeze-dried fruit powder with 10 mL of EtOH:H_2_O (70:30, *v*/*v*). The extraction was performed 3 times during 30 min under stirring. The AHE was centrifuged (10 min at 6000 rpm) and the supernatant was filtered using regenerated cellulose syringe filters (Sartorius Minisart^®^ RC 0.45 µm, Goettingen, Germany). The solvent was then evaporated in a rotary evaporator (Hei-Vap precision, Heidolph, Schwabach, Germany), and the remaining water was removed by lyophilization (Unicryo MC4L, UniEquip, Martinsried, Germany). The obtained residue was redissolved in appropriate solvents at the desired concentrations for further studies.

### 3.3. HPLC-DAD-ESI-MS^n^ Analysis

The chemical characterization of AHE was performed by high performance liquid chromatography coupled with an ion-trap mass spectrometer and a diode array detector. For the study, the freeze-dried AHE was dissolved in MeOH:H_2_O (50:50, *v*/*v*) in a concentration of 30 g/L and filtered using RC syringe filters (Sartorius Minisart^®^ RC 0.45 µm, Goettingen, Germany) previously to injection. The HPLC system (Thermo Electron Corporation, Waltham, MA, USA) consisted of a low-pressure quaternary pump with autosampler and a diode array detector (Finnigan Surveyor Plus, Thermo Fisher Scientific). Separations were carried out on a Phenomenex (Torrance, CA, USA) Gemini-NX C18 column (150 mm × 4.6 mm; 3 µm) and a guard column (4 mm × 3.0 mm). The chromatographic conditions were the following: flow rate 0.4 mL/min, sample injection volume 25 µL, and a binary mobile phase (A, MeOH and B, 0.1% aqueous formic acid). A gradient program was used as follows: 0 to 40 min, linear increase from 10% to 30% of A, 40 to 60 min, increase to 45% of A, 60 to 90 min, linear increase to 100% A and conditions maintained for 5 min; return to initial conditions in 15 min and conditions maintained for 10 min before the next injection. A quadrupole ion-trap mass spectrometer (Finnigan LCQ Deca XP Plus) coupled with an electrospray ionization (ESI) source was used. Data acquisition was performed in positive and negative ion modes in the range 150–1000 *m*/*z*. The interface conditions were applied as follows: capillary temperature, 325 °C; source voltage, 5.0 kV; capillary voltage, −15.0 V for negative mode and 4 V for positive mode; sheath gas (N_2_) flow at 60 arbitrary units and auxiliary gas (N_2_) flow at 23 arbitrary units. Tandem mass spectrometric studies were performed (MS^2^ and MS^3^). For the MS^n^ analyses activation energy of 45% was applied. The pseudomolecular ions were fragmented by collision-induced dissociation (CID) with the nitrogen collision gas in the ion trap. The diode array detection was conducted by scanning between 200 and 600 nm. The identification of the compounds was performed by comparison of the spectral results with commercial standards and literature information Data acquisition and processing was achieved by using Xcalibur software version 2.1.0 (Finnigan, San Jose, CA, USA).

The contents of individual phenolic compounds (expressed in mg per 100 g of açaí powder dry-weight (dw)) were determined by the external standard method using calibration curves of available commercial standards or ones with similar structure: rutin (y = 342703x − 8273, r^2^ = 0.9999, limit of detection (LOD) = 0.16 mg/L and limit of quantification (LOQ) = 0.54 mg/L); catechin (y = 148109x + 895, r^2^ = 0.9999, LOD = 0.10 mg/L and LOQ = 0.34 mg/L); protocatechuic acid (y = 395222x − 1885, r^2^ = 0.9997, LOD = 0.37 mg/L and LOQ = 1.23 mg/L). The anthocyanins abundance in AHE was determined based on the comparison of relative peak areas.

### 3.4. Cell Culture Experiments

Human microvascular endothelial cells (HMEC-1; ATCC, Barcelona, Spain) were used between passages 3 and 7. HMEC-1 were cultured in RPMI 1640 medium supplemented with 10% FBS, 1% penicillin/streptomycin, 1.176 g/L of sodium bicarbonate, 4.76 g/L of Hepes, 1 µg/L of EGF and 1 mg/L of hydrocortisone, and maintained at 37 °C in a humidified 5% CO_2_ atmosphere. Treatments were performed during 16 or 24 h in serum-free conditions. The freeze-dried AHE was dissolved in EtOH:H_2_O (70:30, *v*/*v*) in a concentration of 20 g/L, diluted in serum-free culture medium and added to cell cultures at final concentrations ranging the doses 1 to 75 mg/L. Controls were performed using 0.7% EtOH.

### 3.5. MTS Toxicity Assay

HMEC-1 (2 × 10^5^ cells/mL) were allowed to grow until 70–90% confluence and then incubated with each treatment for 24 h. Cellular viability was assessed using Cell Titer 96^®^ Aqueous ONE Solution Reagent (MTS, [3-(4,5-dimethylthiazol-2-yl)-5-(3-carboxymethoxyphenyl)-2-(4-sulfophenyl)-2H-tetrazolium] colorimetric assay (Promega, Madison, EUA), according to the instructions provided by the manufacturer. Optical density was measured at 492 nm in a microplate reader (Multiskan Ascent, Thermo Electron Corporation, Waltham, MA, USA). Results are expressed as percentage of control, which was considered to be 100%.

### 3.6. BrdU Proliferation Assay

HMEC-1 (2 × 10^5^ cells/mL) were allowed to grow until 70–90% confluence and then incubated with each treatment for 24 h. Cells were treated with the non-cytotoxic concentrations of AHE and incubated with a 5′-bromodeoxyuridine (BrdU) solution and then the in situ detection was performed using an In-Situ Detection Kit (Roche, Amadora, Portugal), according to manufacturer’s instructions. Results are expressed as percentage of control, which was considered to be 100%.

### 3.7. Migration Analysis

To perform injury assay cells were grown to 90% confluence and were scrapped from the culture dish using a pipette tip, leaving a void space. Cells were then incubated for 16 h with the distinct treatments. Cell migration to the damaged area was visualized and photographed on a phase contrast microscope (Nikon, UK), at a magnification of ×200 and quantified using ImageJ software (LOCI, Madison, WI, USA). Results are expressed as percentage of control, which was considered to be 100%.

### 3.8. Invasion Assay

The invasion cell capacity of HMEC-1 in the presence of AHE was then quantified by counting the number of cells that migrated through matrigel-coated transwell (8 µm pore-size; BD-biosciences, San Jose, CA, USA). Cells (5 × 10^4^ cells/mL) were harvested on inserts in serum-free medium and placed on wells containing medium complemented with a chemoattractant (FBS 10%), with the distinct treatments. After incubation for 24 h, membranes were removed from inserts, stained with DAPI-MeOH and visualized under a fluorescence microscope (Zeiss ApoTome, Zaventem, Belgium). Twenty random fields of each membrane were counted, at a magnification of ×200. Results are expressed as percentage of control, which was considered to be 100%.

### 3.9. Matrigel Assay for Evaluation of Capillary-Like Structures Formation

HMEC-1 were incubated with the distinct treatments and added to matrigel-coated plates (BD-biosciences, San Jose, CA, USA). After 16 h of incubation, the number of capillary-like structures was counted in a phase-contrast microscope (Nikon, Surrey, UK), at a magnification of ×200. Each cord portion between ramifications was considered as one capillary-like structure. Results are expressed as percentage of control, which was considered to be 100%.

### 3.10. Evaluation of Antioxidant Capacity in HMEC-1

#### 3.10.1. Superoxide Dismutase Activity

The superoxide dismutase (SOD) activity was evaluated in HMEC-1 lysates (3 × 10^4^ cells/mL) after cell incubation with the distinct experimental conditions. Cell lysis was performed using RIPA buffer and protein content was quantified by Pierce^TM^ BCA protein assay kit (Thermo Fisher Scientific, Waltham, MA, USA). The percentage of inhibition rate of SOD activity was addressed by SOD determination kit (Sigma Aldrich, St. Louis, MO, USA), according to the manufacturer’s instructions. Results are expressed as the % inhibition rate in comparison to control, which was considered to be 100%.

#### 3.10.2. Catalase Activity

Catalase activity was assessed in cell lysates, prepared as described in 2.10.1, with a colorimetric Catalase Activity Assay Kit (Abcam, Cambridge, UK), according to manufacturer instructions. Catalase activity is expressed as nmol/min/mL.

#### 3.10.3. Reactive Oxygen Species Formation

Reactive oxygen species (ROS) generation by HMEC-1, assessed in cell supernatants after treatment with AHE, was performed using 2′,7′-dichlorofluorescin diacetate (DCFDA)-ROS detection kit (DCFDA Cellular ROS Detection Kit, Abcam, UK) according to manufacturer instructions. Results are expressed as means of fluorescence units ± SD.

### 3.11. Statistical Analysis

Results are expressed as the mean ± standard deviation (SD) of three independent experiments. For comparison of the distinct experimental groups, analysis of variance (ANOVA test) followed by the Bonferroni pos-hoc test was used. A difference between experimental groups was considered statistically significant whenever *p* value was <0.05.

## 4. Conclusions

Nowadays, natural products assume a relevant role considering their safety and health-promoting properties. The results here presented clearly demonstrate that açaí fruit freeze-dried powder is a source of angiogenic inhibitors and antioxidant compounds. The chemical characterization of AHE revealed the presence of anthocyanins and flavonoids as major constituents, with the capacity to inhibit endothelial cells migration and the formation of capillary-like structures, with no cytotoxic effects. The anti-angiogenic activity was associated with a reduction of ROS production and an increase of antioxidant enzymatic defenses regulated by SOD and catalase. This is of particular importance concerning the application of AHE for the development of drugs and nutraceuticals as novel therapeutic approaches for angiogenesis-dependent diseases, and also as valuable source of bioactive components for cosmetic products. Nevertheless, further studies should focus on the identification of the main phytochemicals associated with the observed effects among the described molecules, and on the evaluation of its isolated activity as well as possible synergistic effects. Additionally, in vivo studies would have a relevant interest considering possible cytotoxic effects related with the intake of AHE. Further studies may also encompass other commercial açaí products and dietary supplements in order to understand the impact of industrial processing on their chemical profile, bioactivity and health benefits, in comparison to fresh açaí fruit.

## Figures and Tables

**Table 1:** Retention time, maximum absorbance wavelengths and main fragments of anthocyanins tentatively assigned in AHE by HPLC-DAD-ESI-MS^n^, in positive ionization mode. The relative abundance of anthocyanins in AHE is presented as % of total peak area ± standard deviation (SD).

Peak	Rt (Min)	λ_max_ (nm)	[M]^+^ (*m*/*z*)	MS^2^ Fragment Ions (*m*/*z*)	Proposed Identification	Peak Area (%)
4	30.6	280, 517	449	**287**	Cyanidin-3-*O*-glucoside	6.2 ± 0.3
5	34.8	280, 517	595	449, **287**	Cyanidin-3-*O*-rutinoside	89.0 ± 0.3
6	38.7	280, 508	579	433, **271**	Pelargonidin-3-*O*-rutinoside	2.3 ± 0.1
7	41.2	280, 517	609	463, **301**	Peonidin 3-*O*-rutinoside	2.6 ± 0.5

Ions at bold represent base peaks.

**Table 2:** Retention time, maximum absorbance wavelengths and main fragments of non-anthocyanin phenolic compounds tentatively assigned in AHE by HPLC-DAD-ESI-MS^n^, in negative ionization mode. The content of phenolic compounds was determined in mg/100 g dw and expressed as mean ± SD.

Peak	Rt (Min)	λ_max_ (nm)	[M-H]^-^ (*m*/*z*)	MS^2^ Fragments (*m*/*z*)	MS^3^ fragments (*m*/*z*)	Proposed Identification	Content (mg/100 g dw)
*Hydroxybenzoic acids*							
1	18.2	260, 290	153	**109**	-	Protocatechuic acid	9.4 ± 0.9 ^a^
*Proanthocyanidins*							
2	22.5	278	865	739, 713, **695**, 577, 425, 407	-	Proanthocyanidin trimer	6.3 ± 0.1 ^b^
3	23.5	280	577	559, 451, **425**, 407, 289	-	Proanthocyanidin dimer	25.7 ± 0.4 ^b^
						Total	32.0 ± 0.5 ^b^
*Flavonoids*							
8	48.9	277, 343	593	575, 503, **473**, 383, 353	383, **353**	Apigenin-6,8-di-*C*-hexoside (vicenin-2)	4.63 ± 0.2 ^c^
9	49.6	280, 340	579	561, 519, 489, **459**, 429	399, **369**	Luteolin-6-*C*-pentoside-8-*C*-hexoside isomer 1	1.61 ± 0.01 ^c^
10	50.6	280, 340	579	561, 519, 489, **459**, 429	399, **369**	Luteolin-6-*C*-pentoside-8-*C*-hexoside isomer 2	2.47 ± 0.02 ^c^
11	51.4	280, 340	579	561, 519, **489**, 459, 429	399, **369**	Luteolin-6-*C*-hexoside-8-*C*-pentoside	1.40 ± 0.04 ^c^
12	54.4	280, 330	563	545, 503, **473**, 443, 383, 353	383, **353**	Apigenin-6-*C*-pentoside-8-*C*-hexoside	4.1 ± 0.1 ^c^
13	55.8	283, 340	449	287, **269**, 151	241, **225**, 197, 181, 151	Taxifolin deoxyhexose	33.1 ± 0.5 ^c^
14	56.9	270, 348	447	429, 357, **327**	327, **299**, 285	Luteolin-6-*C*-hexoside (orientin)	12.7 ± 0.5 ^c^
15	57.3	270, 346	447	429, 357, **327**	327, **299**, 285	Luteolin-8-*C*-hexoside (isoorientin)	5.4 ± 0.1 ^c^
16	59.2	270, 340	431	341, **311**	**283**	Apigenin-8-*C*-hexoside (vitexin)	4.79 ± 0.05 ^c^
17	62.7	290, 340	287	269, **259**, 243	241, **215**, 173, 151, 125	Dihydrokaempferol	9.6 ± 0.1 ^c^
18	64.1	274, 340	461	371, **341**	313, **298**	Scoparin	5.14 ± 0.02 ^c^
19	66.2	274, 335	689	**609**, 591, 569	519, **489**, 399, 369	Luteolin-*C*-hexoside-*C*-pentosidederivative	11.06 ± 0.05 ^c^
20	68.3	274, 340	533	473, **443**	383, **353**	Apigenin-6,8-di-*C*-pentoside	2.39 ± 0.02 ^c^
21	71.6	275, 330	673	**593**, 575, 503	**473**, 383, 353	Apigenin-di-*C*-hexoside sulfate	11.6 ± 0.2 ^c^
22	75.4	280, 370	301	**179**, 151	**151**	Quercetin	2.54 ± 0.01 ^c^
23	77.2	260, 285, 350	285	**241**, 217, 199, 175, 151, 133	-	Luteolin	2.30 ± 0.06 ^c^
24	79.8	268, 350	315	**300**	283, **271**, 227, 151	Isorhamnetin	5.10 ± 0.04 ^c^
25	80.0	268, 343	329	**314**	**299**	Tricin	3.46 ± 0.08 ^c^
						Total	123.3 ± 0.7 ^c^

Ions at bold represent base peaks; ^a^ identification and quantification performed using authentic standard; ^b^ expressed as catechin equivalents; ^c^ expressed as rutin equivalents.

## Data Availability

The data presented in this study are available within the article.

## Sample Availability

Samples of the compounds are not available from the authors.
